# Effectiveness of empiric carbapenem versus non-carbapenem therapy for extended-spectrum β-lactamase producing Enterobacterales infections in non-intensive care unit patients: a real-world investigation in a hospital with high-prevalence of extended-spectrum β-lactamase producing Enterobacterales

**DOI:** 10.1017/ash.2024.88

**Published:** 2024-06-04

**Authors:** Amy Y. Kang, Mary Elkomos, Danny Pham, Michelle Guerrero, Deborah Kupferwasser, Loren G. Miller

**Affiliations:** 1 Department of Pharmacy Practice, Chapman University School of Pharmacy, Irvine, CA, USA; 2 Department of Pharmacy, Harbor-UCLA Medical Center, Torrance, CA, USA; 3 Division of Infectious Diseases, Lundquist Institute at Harbor-UCLA Medical Center, Torrance, CA, USA; 4 Department of Pharmacy, University of California Irvine Health, Orange, CA, USA; 5 Department of Pharmacy, Memorial Hermann-Texas Medical Center, Houston, TX, USA; 6 Division of Infectious Diseases, Department of Medicine, Harbor-UCLA Medical Center, Torrance, CA, USA

## Abstract

**Objective:**

To investigate whether empiric carbapenem therapy, compared to empiric non-carbapenem therapy, was associated with improved clinical outcomes among hospitalized, non-intensive care unit (ICU) patients with extended-spectrum β-lactamase (ESBL)-producing Enterobacterales infections.

**Methods:**

We performed a retrospective cohort study of adult, non-ICU patients admitted with ESBL-producing Enterobacterales infections. Primary outcome was time to clinical stability from the first empiric antibiotic dose. Secondary outcomes were early clinical response and 30-day all-cause hospital readmission. We used multivariate regression methods to examine time to clinical stability.

**Results:**

Of the 142 patients, 59 (42%) received empiric carbapenems and 83 (58%) received empiric non-carbapenems, most commonly ceftriaxone (49/83, 59%). Median age was 59 years. The most common infection source was urinary (71%). The carbapenem group had a higher proportion of patients who received antibiotics within 6 months of admission (55% vs 28%, *P* < .01) and history of ESBL (57% vs 17%, *P* < .01). There were no significant differences in hours until clinical stability between the carbapenem and non-carbapenem groups (22 (IQR: 0, 85) vs 19 (IQR: 0, 69), *P* = .54). Early clinical response (88% vs 90%, *P* = .79) and 30-day all-cause hospital readmission (17% vs 8%, *P* = .13) were similar between groups.

**Conclusion:**

Among hospitalized non-ICU patients with ESBL-producing Enterobacterales infection, we found no difference in time to clinical stability after the first empiric antibiotic dose between those receiving carbapenems and those who did not. Our data suggest that empiric carbapenem use may not be an important driver of clinical response in patients with less severe ESBL-producing Enterobacterales infection.

## Introduction

Surviving Sepsis Guidelines recommend empiric broad-spectrum antibiotic therapy that covers likely pathogens with subsequent de-escalation once a pathogen is identified.^1^ Early initiation of effective empiric antibiotic treatment has been associated with improved survival, especially in patients with septic shock.^2^ However, in hospitalized, non-intensive care unit (ICU) patients with less severe clinical presentation, it is unclear whether effective empiric therapy leads to significantly improved clinical outcomes. Given the administration of unnecessarily broad antibiotic therapy can drive the emergence of antibiotic-resistant pathogens,^3^ it is important to consider patient-specific risk factors such as severity of acute illness to guide appropriate empiric antimicrobials without excessively broad coverage.

According to the Centers for Disease Control and Prevention Antibiotic Resistance Threats Report, there has been 50% rise in extended-spectrum beta-lactamases (ESBL)-producing Enterobacterales infections between 2012 and 2019, increasing to 197,400 infections in 2019.^4^ At our center, the prevalence of ESBL-producing Enterobacterales has similarly increased which, in turn, has driven further use of empiric carbapenem therapy for patients with infections that could be caused by ESBL-producing Enterobacterales infections. As carbapenem use fuels the emergence of carbapenem-resistant pathogens,^5^ multiple studies have evaluated carbapenem-sparing directed therapy in the setting of ESBL-producing Enterobacterales infections.^6–13^ Notably, in the MERINO trial, a randomized multicenter trial of patients with ESBL-producing *E. coli* and *K. pneumoniae* bloodstream infections, mortality was higher with piperacillin-tazobactam compared to carbapenem therapy.^13^ However, because little is known about the impact of empiric carbapenem therapy among less acutely ill patients with ESBL infections for patients who are hospitalized in non-ICU areas, we investigated whether empiric carbapenem therapy is associated with improved clinical outcomes compared to empiric non-carbapenem therapy among non-ICU patients at a medical center with high prevalence of ESBL-producing Enterobacterales.

## Methods

We performed a retrospective single-center cohort study conducted at a 570 licensed beds public teaching hospital located in Torrance, California. Our hospital has high prevalence of ESBL-producers among Enterobacterales (28% in 2019). For our cohort, we included all adult, hospitalized patients with growth of Enterobacterales in one or more cultures in the hospital Clinical Microbiology Laboratory between 1/1/2019 and 8/31/2020. Patients were excluded from our analysis if: 1) they were admitted to the ICU; 2) antibiotics were started more than 48 hours from the first positive culture; 3) patient had a polymicrobial culture (≥2 different organisms from the same culture); 4) hospital length of stay was <24 hours; 5) positive cultures were felt to reflect colonization (ie, not requiring treatment) based on treating clinician’s assessment; or 6) patient left the facility without completing the treatment against medical advice. This study was approved by the Institutional Review Board at the Lundquist Institute at Harbor-UCLA Medical Center.

Patients were divided into two groups depending on which empiric antibiotic they received: 1) empiric carbapenem therapy, and 2) empiric non-carbapenem therapy. Empiric therapy was defined as antibiotic therapy initiated prior to when the organism’s susceptibilities were known. Patients who received ≥1 dose of a carbapenem as part of empiric therapy were classified as the carbapenem group.

Identification of the presence of ESBL production was determined by the Vitek 2 system (bioMérieux Vitek, Hazelwood, MO) in the hospital’s Clinical Microbiology Laboratory. If an ESBL positive isolates tested susceptible to ceftazidime and ceftriaxone, Clinical and Laboratory Standards Institute disk diffusion method was used to confirm ESBL production by the increased zone diameters (5 mm) in the presence of clavulanate. Antibiotic dosing at the medical center was adjusted by clinical pharmacists based on institutional guidelines.

### Study outcomes

Our primary outcome was days until clinical stability from the first dose of an empiric antibiotic that targeted a gram-negative organism. Clinical stability was determined using the previously defined definitions,^14^ specifically, stabilization of vital signs (temp <37.8°C, HR <100 bpm, RR <24 breaths/min, systolic blood pressure ≥90 mmHg, oxygen saturation 90% or more on room air, and normal mental status (absence of confusion/disorientation)).

We also had two secondary outcomes: early clinical response, and all-cause hospital readmission within 30 days after the end of therapy. Using established definitions,^15,16^ we defined early clinical response as symptomatic improvement without worsening on subsequent days as documented on the treating physician’s progress note plus clinical stability as defined above from receipt of first dose of empiric antibiotic drug for gram-negative organisms to the time until the antibiotic susceptibility report became available. If any of these criteria were not met, patients were classified as lacking early clinical response. Effective antimicrobial therapy was defined as utilization of an agent exhibiting susceptible in vitro activity against the ESBL-producing Enterobacterales based on report from the Clinical Microbiology Laboratory and deemed to be clinically effective based on existing body of literature.

The study investigators trained research assistants to extract data from the Electronic Health Records using a standardized chart abstraction instrument. The investigators confirmed the accuracy of data by allowing research assistants to enter data only after extensive training and demonstrated accuracy. Any discrepancies and ambiguities related to data abstraction were resolved through discussions with research assistants and study investigators.

### Statistical analysis

We used Chi-square test or Fisher's exact test for categorical variables, and student t-test or Mann-Whitney test for continuous variables, as appropriate. To determine factors associated with delayed time to clinical stability, multivariable linear regression was performed. A generalized linear model with the continuous outcome variable, time to clinical stability and pre-defined predictors variables, specifically antibiotic treatment, immunocompromised status, presence of diabetes, and Charlson Comorbidity Index scores,^17^ were assessed. The model also controlled for demographic covariates, specifically age, race/ethnicity, and gender. Model fit was determined by the F-test, and R-squared was evaluated to show the proportion of the total variance explained by the model. Time to clinical stability among patients who were initially unstable was also analyzed visually according to the Kaplan–Meier method. Comparison of the Kaplan-Meier curves was performed using the log-rank test. All statistics were performed using Stata v 17 (College Station, TX), GraphPad v 10.0.0, and SAS software v 9.4 (SAS Institute®, Cary NC).

## Results

**Figure 1.**
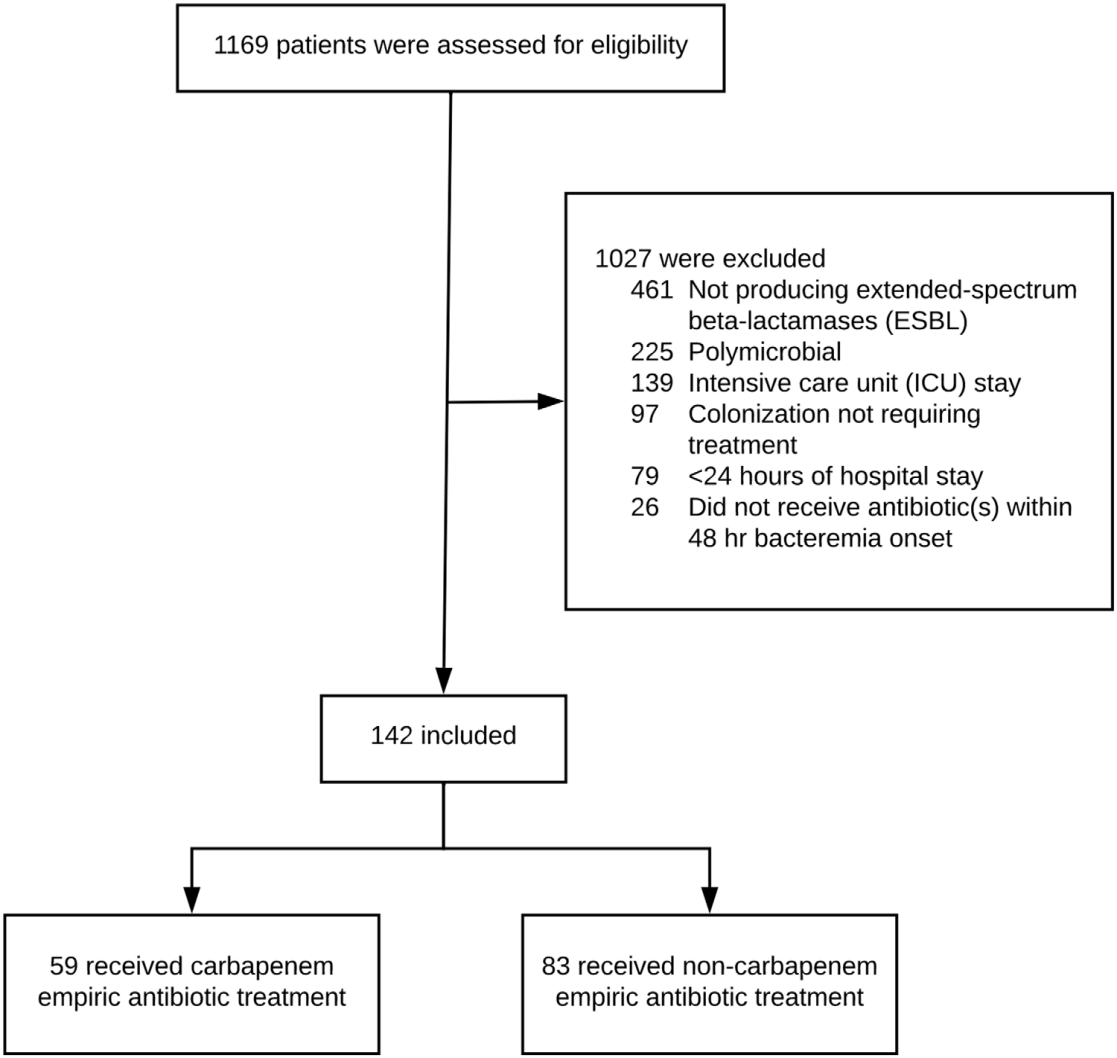
Patient inclusion/exclusion and cohort allocation.

We screened 1169 patient records. Among these patients, 1017 were excluded. Reasons for exclusion included non-ESBL-producing Enterobacterales (n = 461), polymicrobial infections (n = 225), and ICU stay (n = 139) (Figure 1). In total, 142 patients met study criteria and were included in the analysis. Fifty-nine patients (42%) received empiric carbapenem therapy and 83 (58%) received empiric non-carbapenem therapy.

The mean age for included patients was 59 years (SD ± 18) and 48% of patients were male. In terms of differences between the carbapenem group and the non-carbapenem group, the empiric carbapenem therapy group had a significantly higher proportion of patients who previously received antibiotics within 6 months of admission (56% vs 27%, *P* < .01), and had a higher history of ESBL-producing Enterobacterales infections or colonization (56% vs 16%, *P* < .01), and higher percentage of a chronic indwelling Foley catheter (24% vs 6%, *P* < .01) (Table 1). Clinical presentations were similar between the two groups except the empiric carbapenem group had higher temperatures (median 38 IQR (37, 39) vs 37 (37, 39), *P* < .01) (Table 1).

**Table 1.** Baseline characteristics and clinical presentations

	Carbapenem (n = 59)	Non-carbapenem (n = 83)	*P* value
Male, no. (%)	29 (49)	39 (47)	.80
Age, mean (±SD)	58 (±17)	60 (±18)	.62
Hispanic or Latino, no. (%)	37 (63)	59 (71)	.29
**Race, no. (%)**			
American Indian or Alaskan Native	0 (0)	1 (1)	.99
Asian	3 (5)	1 (1)	.31
Black	6 (10)	7 (8)	.77
European/White	6 (10)	8 (10)	.99
Latin American	40 (68)	61 (73)	.46
Unknown/Other	4 (7)	5 (6)	.99
**Admission source, no. (%)**			
Home or apartment	49 (83)	75 (90)	.20
LTCF/SNF	6 (10)	6 (7)	.56
Homeless	3 (5)	1 (1)	.31
Outside Hospital	1 (2)	1 (1)	.99
Previous hospitalization within 6 months of current admission, no. (%)	27 (46)	28 (34)	.15
Recent surgery/procedure within 6 months of admission, no. (%)	14 (24)	17 (20)	.64
Previous antibiotics within 6 months of admission, no. (%)	33 (56)	22 (27)	**<.01**
Prior history of ESBL-producing Enterobacterales Infections/colonization, no. (%)	33 (56)	13 (16)	**<.01**
Immunocompromised, no. (%)	10 (17)	6 (7)	.08
HIV	1 (2)	1 (1)	.99
Chemotherapy within 6 months	6 (10)	4 (5)	.32
Absolute neutrophil count ≤100 cells/µL	0	1 (1)	.99
Immunomodulatory therapy or corticosteroids for ≥14 days	3 (5)	1 (1)	.31
ESRD on HD, no. (%)	5 (8)	9 (11)	.64
ESLD/cirrhosis, no. (%)	2 (3)	8 (10)	.15
Structural lung diseases, no. (%)	3 (5)	5 (6)	.81
Diabetes, no. (%)	35(59)	39 (47)	.15
Chronic indwelling foley, no. (%)	14 (24)	5 (6)	**.01**
Charlson Comorbidity Index, median (IQR)	3 (2, 5)	4 (2, 6)	.09
qSOFA Score, median (IQR)	1 (0, 1)	1 (0, 1)	.85
Source of infection^a^, no (%)			
Urinary	46 (78)	55 (66)	.13
Bacteremia	13 (22)	12 (14)	.27
Intra-abdominal	3 (5)	9 (11)	.36
Skin and soft tissue	2 (3)	11 (13)	.07
Bone and joint	1 (2)	2 (2)	.99
Pneumonia	2 (3)	0	.17
Community-acquired, no. (%)	54 (92)	66 (80)	.05
ESBL-producing Enterobacterales isolated from the culture, no. (%)			
*E. coli*	50 (85)	76 (92)	.21
*Klebsiella pneumoniae*	6 (10)	6 (7)	.56
*Proteus*	3 (5)	1 (1)	.31
Source control needed, no. (%)	7 (12)	17 (20)	.18
Source control achieved, no. (%)	7 (100)	16 (94)	
Drainage of abscess	0 (0)	7 (8)	.04
Removal of venous catheter/lines	1 (2)	1 (1)	.99
Removal of urinary catheter	5 (8)	3 (4)	.28
Other surgery/procedure	1 (2)	5 (6)	.40
Temperature, median (IQR)	38 (37, 39)	37 (37, 38)	**.01**
Lactate, mmol/L, median (IQR)	1.6 (1.1, 2.9)	1.5 (1.0, 4.0)	.69
White blood cell, ×10^9^/L, median (IQR)	12.3 (8.5, 17.1)	10.1 (7.7, 14.0)	.11
Serum creatinine, mg/dL, median (IQR)	1.3 (0.8, 1.9)	1.1 (0.8, 2.3)	.75

Abbreviations: LTCF/SNF, long-term care facilities/skilled nursing facilities; ESBL, extended-spectrum β-lactamase; ESRD, end-stage renal disease; ESLD, end-stage liver disease; qSOFA, quick sequential organ failure assessment.

a Some patients had more than one source of infection.

**Figure 2.**
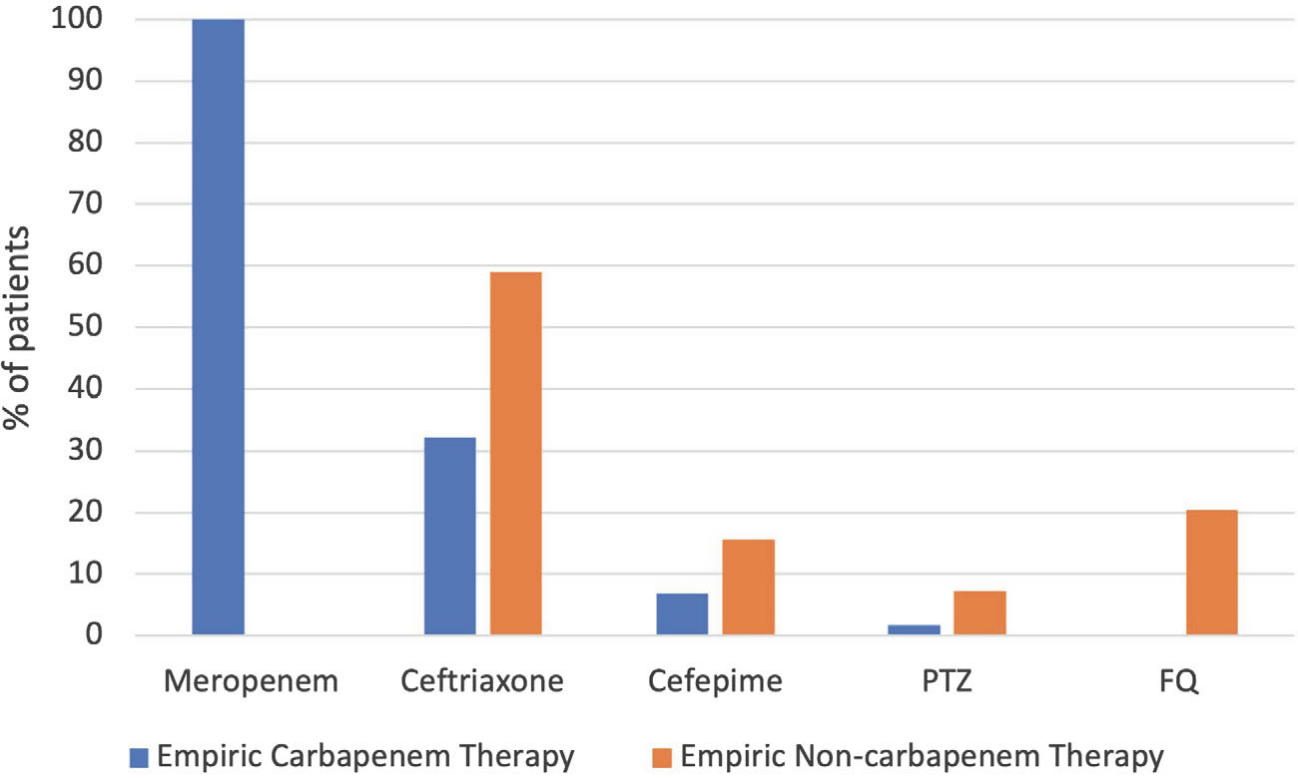
Empiric therapy for ESBL-producing Enterobacterales in non-ICU settings. Abbreviations: ESBL, extended-spectrum beta-lactamase; PTZ, piperacillin-tazobactam; FQ, fluoroquinolones; SMX/TMP, sulfamethoxazole-trimethoprim. Note that some patients received more than one antibiotic as empiric therapy.

The most common source of infection was urinary for both empiric carbapenem and non-carbapenem groups (78% vs 66%, *P* = 0.13) and both groups had similar amounts of bloodstream infections (22% vs 14%, *P* = 0.27). For empiric therapy, among patients in the carbapenem group, all 59 (100%) received meropenem. Among patients in the non-carbapenem group, ceftriaxone was the most common therapy administered (59% (49/83), followed by fluoroquinolones (20% (17/83)) and cefepime (16% (13/83)) (Figure 2). All patients (100%) in the carbapenem group received effective empiric therapy, but only 28% (23/83) of patients in the non-carbapenem group received effective empiric therapy (based on *in vitro* susceptibilities) (Table 2). Of note, the median number of carbapenem doses given as an empiric therapy until susceptibilities resulted was 5 (IQR 2, 8).

**Table 2.** Treatment

	Carbapenem (n = 59)	Non-Carbapenem (n = 83)	*P* value
Effective empiric therapy based on *in vitro* cultures and susceptibilities (%)	59 (100)	23 (28)	<.01
Time to effective therapy, median hours (IQR)	3 (0.1, 17)	53 (35, 75)	<.01
Inpatient antibiotic days of therapy, median days (IQR)	5 (4, 7)	5 (3, 8)	.45
Total duration of therapy in days, median (IQR)	9 (7, 12)	9 (7, 12)	.79

**Figure 3.**
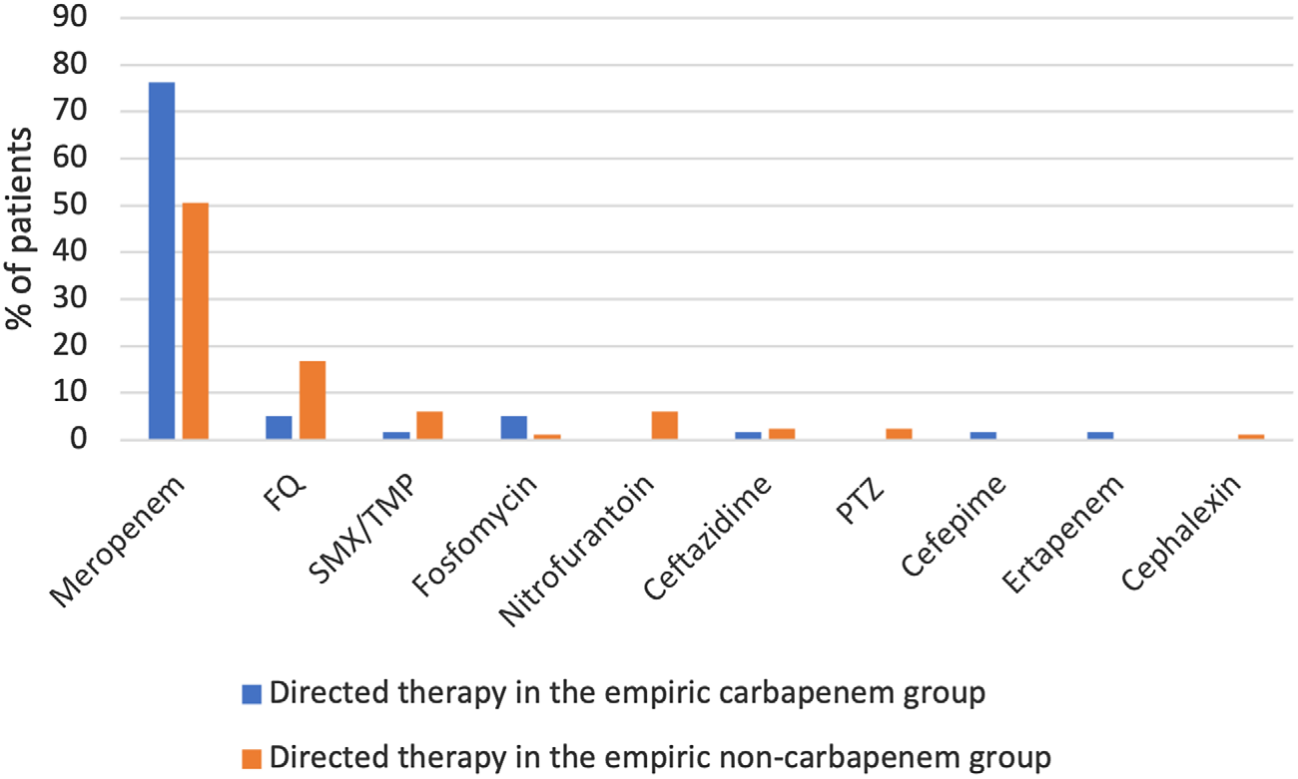
Directed therapy. Abbreviations: FQ, fluoroquinolones; SMX/TMP, sulfamethoxazole-trimethoprim; PTZ, piperacillin-tazobactam.

For directed therapy, in the carbapenem group, the majority of patients received a carbapenem 76% (45/59) and in non-carbapenem group 51% (42/83) received carbapenems. Common non-carbapenem directed therapies include fluoroquinolones (5% (3/59) in the carbapenem group and 17% (14/83) in the non-carbapenem group), fosfomycin (5% (3/59) in the carbapenem group and 1% (1/83) in the non-carbapenem group), and trimethoprim/sulfamethoxazole (2% (1/59) in the carbapenem group and 6% (5/83) in the non-carbapenem group) (Figure 3).

Time to effective therapy was significantly shorter in the carbapenem group compared with the non-carbapenem group (median 3 hours vs 53 hours, *P* < 0.01) (Table 2). Inpatient antibiotic days of therapy were similar in the carbapenem and non-carbapenem groups, (median 5 days (IQR: 4, 7) vs 5 days (IQR: 3, 8), respectively, *P* = 0.45) as were total duration of therapy (9 days (IQR: 7, 12) vs 9 days (IQR: 7, 12), *P* = 0.79). Most patients were prescribed antimicrobial therapy upon discharge (71% in the carbapenem group vs 63% in the non-carbapenem group, *P* = 0.29).

Our primary outcome, time to clinical stability, was similar in the carbapenem and non-carbapenem groups (22 hours (IQR: 0, 85) vs 19 hours (IQR: 0, 69, *P* = 0.54). Similarly, our secondary outcomes, early clinical response (88% vs 90%, *P* = 0.78), and 30-day all-cause hospital readmission (17% vs 8%, *P* = 0.13), were similar between the two groups (Table 3). Finally, 30-day mortality was similar between groups: 3% (2/59) in the carbapenem and 2% (2/83) in the empiric non-carbapenem group, *P* > 0.99).

**Table 3.** Clinical outcomes

	Carbapenem (n = 59)	Non-carbapenem (n = 83)	*P* value
Hours until clinical stability, median (IQR)	22 (0, 85)	19 (0, 69)	.54
Early clinical response, no. (%)	52 (88)	75 (90)	.79
30-day all-cause hospital readmission, no. (%)	10 (17)	7 (8)	.13

Our multivariate regression model found that significant predictors of delayed time to clinical stability were age (regression coefficient = −1.7; *P* = 0.01), American Indian or Alaskan Native race (regression coefficient = 218; *P =* 0.03), and Charlson Comorbidity Index scores (regression coefficient = 14; *P =* 0.003). Of note, our model explained 15% (*R* ^2^ = 0.15) of the variability for time to clinical stability.

**Figure 4.**
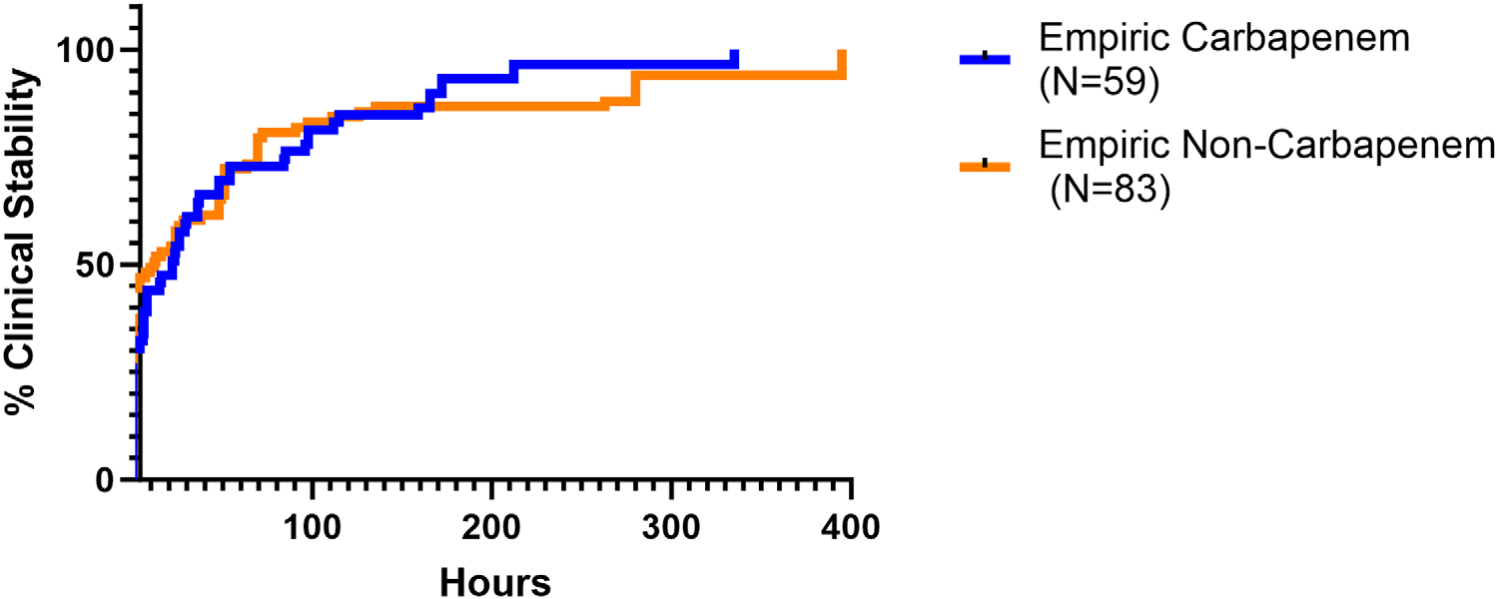
Kaplan–Meier plot of the proportion of patients who presented clinically unstable.

Kaplan-Meier analysis of the subset of patients who presented clinically unstable at the start of the study (n = 94) found the probability of achieving clinical stability between the treatment groups was not significantly different (*P* = 0.14) (Figure 4).

## Discussion

In our study of 142 patients who were hospitalized in non-ICU settings with ESBL Enterobacterales infections, we found that those treated with empiric non-carbapenem therapy had similar time to clinical stability, comparable early clinical response, and 30-day all-cause hospital readmission compared with patients who received empiric carbapenem therapy. Our findings suggest that the superiority of carbapenem therapy over non-carbapenem therapy for ESBL Enterobacterales infections may be limited to more severely ill, ICU patients.^13^

Empiric therapy of Enterobacterales remains challenging. The rapidly increasing prevalence of ESBL-producers among Enterobacterales,^18^ coupled with nonspecific risk factors for ESBL production (e.g. indwelling urinary catheters, history of recurrent UTIs, recent antimicrobial use, long hospital stay, or malignancy), likely drives increasingly heavy use of carbapenem as empiric therapy for wide variety of patients with possible ESBL-producing Enterobacterales infections. But carbapenem overuse itself has major downsides as carbapenem overuse may further drive the emergence of carbapenem-resistant Enterobacterales. Given concerns about carbapenem overuse and resultant carbapenem-resistant Enterobacterales, previous studies investigated carbapenem-sparing therapy for ESBL infections. However, majority of these studies focused on directed therapy once the organism has been identified and microbiology susceptibility has been released.^6–12^

Clinical investigations of empiric therapy in hospitalized patients with ESBL Enterobacterales infections have shown conflicting results on patient outcomes. One meta-analysis concluded that patients with bacteremia due to ESBL-producing Enterobacterales found no significant difference in mortality between patients given empiric β-lactam/β-lactamase inhibitors compared and those given empiric carbapenems.^19^ However, the investigators acknowledged that patient populations were heterogeneous, and results may have been subjected to selection bias due to clinicians choosing to administer carbapenems in sicker patients, biasing the meta-analysis towards being unable to detect differences between groups. In contrast, our investigation only included hospitalized patients not admitted to ICUs. Our lack of finding improved outcomes in the carbapenem group suggests that, similar to the aforementioned meta-analysis, the assertion that empiric antibiotic therapy may be less important in clinical improvement in less severely ill patients. Clearly, there are clinical benefits of hospitalization for infection besides antimicrobial therapy such as fluid resuscitation, and correcting hypoxemia and electrolyte disorders, among other interventions.^20^

A propensity-weighted multicenter cohort study evaluated efficacy of empiric carbapenem vs. non-carbapenem in patients with septic shock from urinary ESBL-producing Enterobacterales. The investigators found that empiric non-carbapenem therapy was not associated with significantly higher mortality compared with a carbapenem regimen.^21^ However, it should be noted that urinary tract infections are generally associated with lower mortality rates^22^ and study may have been underpowered to detect mortality differences as numerical differences in mortality ((10/69 (15%) in carbapenem group vs 6/87 (7%) in non-carbapenem group, *P* = 0.16) may suggest that larger cohorts may have adequate power to detect differences. Our investigation focused on hospitalized patients who are at non-ICU level of care, and, similarly, we also did not observe a signal of improved clinical outcomes in empiric carbapenem group. Another study evaluating the clinical outcomes of patients with acute pyelonephritis caused by ESBL-producing Enterobacteriales found that inappropriate empirical antibiotic therapy did not significantly increase treatment failure rates. While this finding helps in decreasing unnecessary empiric use of carbapenems for acute pyelonephritis, this relationship between empiric antibiotic therapy and patient outcomes may not apply to other infection sites. Our study had only a minority of patients whose infection source was non-urinary (29%); thus our findings may be more relevant to urinary tract infections compared to non-urinary tract infections. While it is possible that initial inappropriate therapy for ESBL-producing Enterobacterales infections from a non-urinary source may not adversely affect clinical outcomes for patients who are not severely ill, given we cannot exclude the possibility that in non-urinary ESBL-producing Enterobacterales infections, empiric therapy with carbapenems could be superior to non-carbapenems.

In our multivariable regression analysis, one of the factors significantly associated with longer time to clinical stability was having higher Charlson Comorbidity Index. Such a finding is not surprising. Comorbidities have been shown to be the significant factor not only for the delayed clinical recovery but also for readmission and/or increased mortality.^23,24^ As such, patients with significant comorbidities with ESBL-producing Enterobacterales infections may require extra attention given their propensity to have delayed time to clinical stability, regardless of the appropriateness of empiric antibiotics.

Our study has limitations. First, it was a single-center, retrospective cohort with a relatively modest sample size. Nevertheless, our primary outcome, hours until clinical stability had similar numerical values suggesting that a larger sample size would be unlikely to find differences that could not be detected. Second, as we followed treating clinicians’ determination in including patients who were deemed to have true infection (e.g. requiring antibiotic treatments), some patient's ESBL-producing Enterobacterales isolates may have represented colonization or contamination. However, patients in our cohort presented with signs and symptoms of infection, suggesting that most had true infections. Third, the majority of infections in our study were urinary without concomitant bacteremia. Thus our results may not be applicable for patients with more invasive infections who do not require ICU-level of care. Fourth, we were only able to capture hospital readmissions within our medical network (the Los Angeles County Department Health Services (DHS)). We may have underestimated hospital readmission given patients may have been re-hospitalized outside the DHS system. However, our medical center is part of safety-net system, so patient’s willingness and ability to receive care at outside hospitals may have been limited.

In conclusion, among hospitalized non-ICU patients with ESBL-producing Enterobacterales infection, we found no difference in clinical outcomes between those receiving empiric carbapenems and those who did not. Our data suggest that empiric carbapenem use may not be an important driver of clinical response in patients with less severe ESBL-producing Enterobacterales infection. If we found a true relationship, our findings may have important antimicrobial stewardship implications and could contribute to the argument that infections among hospitalized patients forgoing empiric carbapenem therapy among non-ICU patients do not add significant risks of poor clinical outcomes. Given the rising concerns of carbapenem-resistant Enterobacterales infections, clinicians should consider non-carbapenem antibiotics for empiric therapy of clinically stable patients without life-threatening infections. Future investigations involving larger and more diverse patient populations are needed to confirm our findings.
